# Inter-Antibody Variability in the Clinical Pharmacokinetics of Monoclonal Antibodies Characterized Using Population Physiologically Based Pharmacokinetic Modeling

**DOI:** 10.3390/antib13030054

**Published:** 2024-07-09

**Authors:** Mokshada Kumar, Sravani Lanke, Alka Yadav, Mfonabasi Ette, Donald E. Mager, Dhaval K. Shah

**Affiliations:** 1 Department of Pharmaceutical Sciences, School of Pharmacy and Pharmaceutical Sciences, The State University of New York at Buffalo, Buffalo, NY 14214-8033, USA; mokshada@buffalo.edu (M.K.); sravanil@buffalo.edu (S.L.); alkachan@buffalo.edu (A.Y.); mfonabas@buffalo.edu (M.E.); dmager@buffalo.edu (D.E.M.); 2 Enhanced Pharmacodynamics, LLC, Buffalo, NY 14203, USA

**Keywords:** antibody, clinical pharmacology, pharmacokinetics, PBPK model

## Abstract

The objective of this work was to develop a population physiologically based pharmacokinetic (popPBPK) model to characterize the variability in the clinical PK of monoclonal antibodies (mAbs) following intravenous (IV) and subcutaneous (SC) administration. An extensive literature search was conducted and clinical PK data for FDA-approved as well as non-approved mAbs were collected. Training and validation datasets of 44 and 9 mAbs exhibiting linear pharmacokinetics were used for model development. The variability in antibody PK was captured by accounting for different rate constants of pinocytosis (CL_up_) and intracellular degradation (k_deg_) for different mAbs. Typical values for CL_up_ and k_deg_ and their respective inter-antibody variabilities ($\omega_{Clup}$, $\omega_{Kdeg})$ were estimated to be 0.32 L/h/L and 26.1 $h^{-1}$ (73% and 46%). Varied absorption profiles following SC dosing were characterized by incorporating inter-antibody variability in local degradation (k_SC_) and rate of lymphatic uptake (S_Lu) of mAbs. Estimates for typical k_SC_ and S_Lu values, and $\omega_{Ksc},\omega_{S\_Lu},$ were found to be 0.0015 $h^{-1}$ and 0.54 (193%, and 49%). FDA-approved mAbs showed less local degradation (0.0014 $h^{-1}$ vs. 0.0038 $h^{-1}$) compared with other clinically tested mAbs, whereas no substantial differences in physiological processes involved in disposition were observed. To evaluate the generalizability of estimated PK parameters and model validation, the final popPBPK model was used to simulate the range of expected PK for mAbs following SC administration of nine different mAbs that were not used for model-building purposes. The predicted PK of all nine mAbs was within the expected range specified *a priori*. Thus, the popPBPK model presented here may serve as a tool to predict the clinical PK of mAbs with linear disposition before administering them to humans. The model may also support preclinical-to-clinical translation and ‘first-in-human’ dose determination for mAbs.

## 1. Introduction

Monoclonal antibodies (mAbs) have revolutionized the field of medicine in the last couple of decades. Their ability to provide targeted therapy with fewer off-target effects, long half-life, reduced immunogenicity, and less propensity for drug–drug interactions make them highly desirable therapeutic modalities. A total of 155 mAbs have been approved by the FDA up until 2022, with a significant increase in FDA approvals post-2013 [1]. This versatile modality has found application in the treatment of almost all types of diseases and has also provided relief to patients suffering from diseases with few treatment options such as infectious diseases (e.g., Ebola), refractory cancers (e.g., refractory diffuse large B-cell lymphoma), and rare diseases (e.g., eosinophilic granulomatosis with polyangiitis) [2]. Advances in protein engineering and antibody discovery technologies (e.g., phage display, yeast display, affinity maturation, antibody humanization, etc.) have significantly expedited the discovery and development of antibody-based therapies, with more than twenty submissions to regulatory agencies every year. With such a fast pace of discovery, it is becoming increasingly important to expedite the preclinical evaluation and clinical translation of these modalities. An objective and quantitative way to accomplish this is to employ the model-informed drug development (MIDD) strategy [3,4], which can not only help characterize and predict the pharmacokinetics (PK) and pharmacology of drug molecules but can also provide insights into the variability associated with these properties using learn and confirm paradigms. To facilitate the application of this quantitative strategy for mAb development, we have developed a population-based PK model that can characterize and predict the PK and associated variability of mAbs in the clinic.

Even though therapeutic mAbs follow some general patterns of absorption and disposition based on their shared size and structure, individual mAbs have diverse PK profiles post-administration. Antibody half-lives ranging from a few days to more than three weeks have been reported in the clinic. Varying binding affinities to FcRn and dose-dependent target-mediated drug disposition are considered primary sources of this variability [5]. Antibody binding to FcRn regulates its ability to recycle and escape catabolic clearance, and different FcRn affinities can introduce inter-antibody variability in systemic drug exposure. Target-mediated drug disposition introduces significant variability in PK as it depends on multiple factors like receptor expression, receptor internalization rate, antibody binding affinity, and dose [6]. Pharmacokinetic variability has also been observed for mAbs that have similar affinity to FcRn and exhibit linear pharmacokinetics. There is a dearth of knowledge in the literature regarding the extent of this diversity in the clinic. Whereas some studies have compiled non-compartmental analysis (NCA) and compartmental model-derived parameters, such as clearance and volume of distribution for different mAbs and their variabilities [7,8], a comprehensive analysis of inter-antibody variability using a physiologically based PK (PBPK) model is lacking.

PBPK models mathematically describe the physiological processes involved in drug absorption and disposition. They can provide a platform to characterize antibody PK in the clinic and investigate the mechanisms involved in the absorption, distribution, and elimination variability between mAbs [9]. In contrast, population PK models are regularly used to characterize the variability in drug exposure amongst study subjects and patients [10]. However, population PBPK models have not been used to capture the inter-antibody variability in clinical PK to date. Previously, a minimal PBPK model was used to obtain a better understanding of the clinical PK of mAbs [11]. However, this approach can include empirical clearance and distribution parameters and by its definition excludes a whole-body description of drug disposition.

Therefore, in this study, data from all clinically tested mAbs (approved and investigational) were used to develop a population physiologically based pharmacokinetic (popPBPK) model that provides insights into the general pharmacokinetic behavior of mAbs with linear PK as well as their variability observed in the clinic. We hypothesize that the inter-antibody variability in disposition stems from variability in physiological processes of pinocytic uptake by endothelial cells and lysosomal degradation of mAbs and that absorption comes from variability in lymphatic uptake and local degradation. An exhaustive database of published clinical PK profiles was first established from all clinically tested mAbs with linear pharmacokinetics post intravenous (IV) and subcutaneous (SC) administration. The database was used to develop a popPBPK model by characterizing the inter-antibody variability in key physiological processes mentioned above. The established model was then used to simulate a range of possible PK profiles in the clinic for IV and SC administration. This range was then externally validated with a separate dataset of antibody PK profiles following SC administration.

## 2. Methods

### 2.1. mAb Clinical PK Data Collection

All published clinical PK data for mAbs were collected from the literature. Extensive lists of therapeutic mAbs from The Antibody Society were referenced to find all published clinical PK mAb data. A thorough search was conducted using online resource platforms like Google Scholar, Web of Science, Elsevier, and PubMed with the keywords antibody drug name, pharmacokinetics, single dose, and clinical PK. Pharmacokinetic data on FDA-approved mAbs were also obtained from BLA review documents available on the official FDA website: www.accessdata.fda.gov (accessed on 1 January 2023). The majority of data obtained were from Phase 1 studies spanning multiple dose levels post intravenous and subcutaneous administration. PK data solely consisting of trough concentrations were available from multiple dose studies but were excluded owing to the lack of time points to confidently estimate parameters. Only data from the first cycle of multiple dosing studies were incorporated in the analysis. All published PK profiles were digitized online using the free web tool Webplot digitizer [12] to build the database. The PK profiles in our database were dose-normalized to identify and exclude mAbs that exhibit nonlinear pharmacokinetics due to target-mediated drug disposition (TMDD). Any antibody with published proof of dose-dependent PK in the literature was also excluded from the current analysis to focus on variability stemming from linear disposition pathways. For mAbs like infliximab, which are known to elicit substantial immunogenicity in the body, mean PK profiles from non-immunogenic patients were incorporated in the analysis. The PK profiles were further classified as ‘FDA Approved’ or ‘clinically tested’ to explore possible differences in PK as a factor influencing mAb approval. For the purposes of this text, ‘FDA approved’ refers to mAbs that have been approved by the FDA, and ‘clinically tested’ mAbs refer to mAbs that have been tested in the clinic but have not yet received FDA approval. Non-compartmental analysis of digitized concentration–time profiles was conducted using the ‘PKNCA’ package in R.

### 2.2. Model Structure

**Figure 1:**
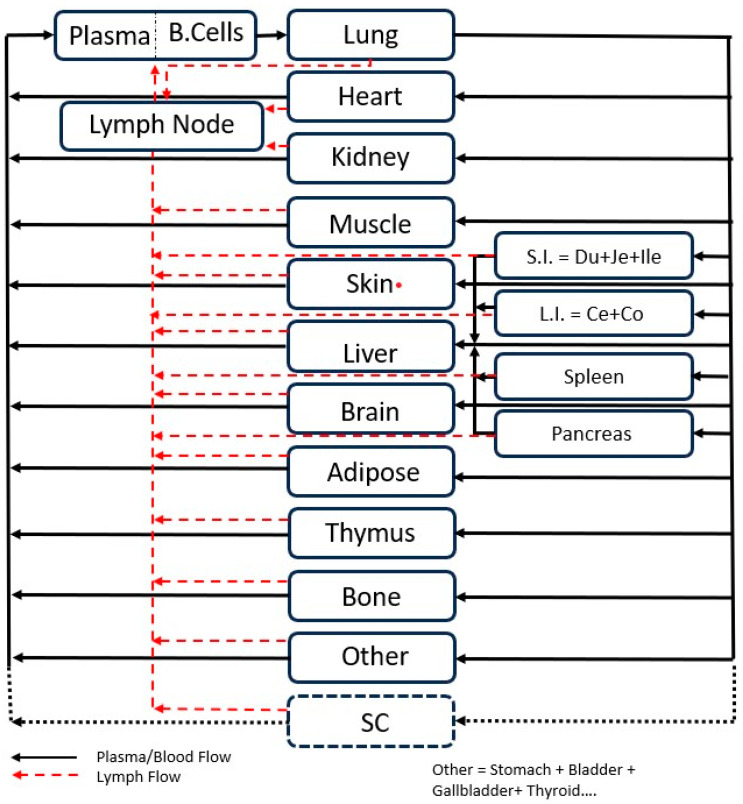
Model structure of the whole body PBPK model for mAbs. Each compartment refers to a specific organ in the body. The remaining organs not represented as separate compartments have been lumped into the ‘Other’ compartment. All compartments are interconnected physiologically via lymph and blood flow. The SC compartment was added to capture PK data post-SC administration. IV dose is administered into the plasma compartment and the SC dose is administered into the SC compartment. S.I., L.I., Du, Je, Ile, Ce, and Co represent small intestine, large intestine, duodenum, jejunum, ileum, cecum, and colon.

The PBPK model was adapted from Shah and Betts [9]. Briefly, the model consists of blood and fifteen tissues, namely lung, liver, heart, kidney, spleen, muscle, skin, adipose, bone, thymus, small intestine, large intestine, pancreas, lymph node, and ‘other’ compartments. All tissues are physiologically interconnected via blood and lymphatic flow as shown in Figure 1.

**Figure 2:**
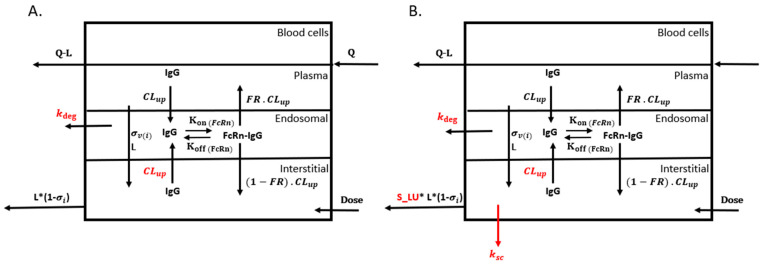
Tissue-level PBPK model structure. Structure of all tissues (except lymph node and SC) (**A**) and the SC tissue space (**B**). Inter-antibody variability was assigned to k_deg_ and CL_up_ for IV mAbs and to k_deg_, CL_up_, S_LU, and k_SC_ for SC-administered mAbs.

Each tissue, apart from the lymph node, is further made up of vascular (plasma and blood cells), endosomal, interstitial, and cellular sub-compartments, as shown in the tissue-level model structure in Figure 2. In summary, mAbs enter the tissue via arterial blood flow. These mAbs can then undergo pinocytosis (CL_up_) into an endosomal sub-compartment or are transported into the interstitial sub-compartment via convection (L). The model assumes that FcRn is only present in the endosomal compartment of endothelial cells lining the blood vessels. Thus, the mAbs present in the endosomal compartment can either interact with FcRn to form an ‘FcRn-mAb’ complex via binding parameters ($K_{on}^{FcRn}$ and $K_{off}^{FcRn}$) or undergo non-specific clearance (k_deg_). The FcRn-bound antibody is recycled (FR) into the plasma or exocytosed (1-FR) into the interstitial compartment. The antibody in the interstitial compartment can either be endocytosed into the endosomal compartment or transported to the lymph node via convective flow (L). The resistance to convective flow from vascular to interstitial tissue and interstitial tissue to a lymph node is represented by vascular ($\sigma_{v}$) and interstitial ($\sigma_{i}$) reflection coefficients. The mAbs used in the current analysis only display linear pharmacokinetics, thus antigen binding in the tissue-level model was not included.

### 2.3. SC Model Structure

The SC model structure was adapted from a previously published model [13]. To capture the PK of mAbs administered subcutaneously, the skin was divided into 2 separate compartments—a subcutaneous compartment (a smaller part of the skin where the dose is administered) and the ‘rest of the skin’ compartment. The subcutaneous tissue, like other tissues, is also subdivided into 4 sub-compartments—vascular, endothelial, interstitial, and cellular—and has a similar structure to other tissues (Figure 2). The subcutaneous dose is administered in the interstitial region of the subcutaneous compartment. Additionally, a first-order degradation rate (k_SC_) was introduced in the SC interstitial compartment to account for local degradation near the injection site. A scaling factor, S_LU, was also multiplied by the convective flow of antibodies from the interstitial region to the lymph node compartment. S_LU is an antibody-specific parameter and accounts for differences in driving forces for lymphatic uptake of the antibody based on the local distribution and nonspecific interaction of antibodies at the injection site.

### 2.4. Model Equations

The model equations for the PBPK model used to capture IV data are the same as previously published [9]. The general equations for the SC model that have been modified from the IV model are:

Central Blood Compartment

Plasma

$$\begin{array}{l} \frac{{dC}_{pl}}{dt}=(\left(Q_{he}-L_{he}\right).C_{he}^{V}+\left(Q_{ki}-L_{ki}\right).C_{ki}^{V}+\left(Q_{mu}-L_{mu}\right).C_{mu}^{V}+\left(Q_{sk}-L_{sk}\right).C_{sk}^{V}+\left(Q_{SC}-L_{SC}\right).C_{SC}^{V}+((Q_{liv}-\\ L_{liv})+(Q_{sp}-L_{sp})+(Q_{pa}-L_{pa})+(Q_{SI}-L_{SI})+(Q_{LI}-L_{LI})).C_{liv}^{V}+\left(Q_{br}-L_{br}\right).C_{br}^{V}+\left(Q_{ad}-L_{ad}\right).C_{ad}^{V}+(Q_{th}-\\ L_{th}).C_{th}^{V}+\left(Q_{bo}-L_{bo}\right).C_{th}^{V}+\left(Q_{oth}-L_{oth}\right).C_{oth}^{V}+L_{LN}.\;C_{LN}-\left(Q_{lu}.C_{pl}\right))/V_{pl} \end{array}$$

Blood cells

$$\frac{dC_{BC}}{dt}=(Q_{he}.C_{he}^{BC}+Q_{ki}.C_{ki}^{BC}+Q_{mu}.C_{mu}^{BC}+Q_{sk}.C_{sk}^{BC}+Q_{sc}.C_{SC}^{BC}+\left(Q_{liv}+Q_{sp}+Q_{pa}+Q_{SI}+Q_{LI}\right).C_{liv}^{BC}\;\;\;\;\;\;\;\;\;\;\;\;\;\;\;\;\;\;+Q_{br}.C_{br}^{BC}+Q_{ad}.C_{ad}^{BC}+Q_{th}.C_{th}^{BC}+Q_{bo}.C_{bo}^{BC}+Q_{oth}.C_{oth}^{BC}-\left(Q_{lu}.C_{BC}\right))/V_{BC}$$

Lymph Node

$$\begin{array}{ll} \frac{{dC}_{LN}}{dt}=(\left(1-\sigma_{lu}^{I}\right). & L_{lu}.C_{lu}^{I}+\left(1-\sigma_{he}^{I}\right).L_{he}.C_{he}^{I}+\left(1-\sigma_{ki}^{I}\right).L_{ki}.C_{ki}^{I}+\left(1-\sigma_{mu}^{I}\right).L_{mu}.C_{mu}^{I}+\left(1-\sigma_{sk}^{I}\right).L_{sk}.C_{sk}^{I}\\ & +\left(1-\sigma_{SC}^{I}\right).L_{SC}.C_{SC}^{I}+\left(1-\sigma_{LI}^{I}\right).L_{LI}.C_{LI}^{I}+\left(1-\sigma_{SI}^{I}\right).L_{SI}.C_{SI}^{I}+\left(1-\sigma_{sp}^{I}\right).L_{sp}.C_{sp}^{I}+\left(1-\sigma_{pa}^{I}\right).L_{pa}.C_{pa}^{I}\\ & +\left(1-\sigma_{liv}^{I}\right).L_{liv}.C_{liv}^{I}+\left(1-\sigma_{br}^{I}\right).L_{br}.C_{br}^{I}+\left(1-\sigma_{ad}^{I}\right).L_{ad}.C_{ad}^{I}+\left(1-\sigma_{th}^{I}\right).L_{th}.C_{th}^{I}\\ & +\left(1-\sigma_{bo}^{I}\right).L_{bo}.C_{bo}^{I}+\left(1-\sigma_{oth}^{I}\right).L_{oth}.C_{oth}^{I}-L_{LN}.C_{LN})/V_{LN} \end{array}$$

The model equations for all tissues other than the interstitial sub-compartment of subcutaneous tissue are the same as those of the IV model.

Subcutaneous tissue interstitial compartment

$$\frac{{dC}_{SC}^{I}}{dt}=(\left(1-\sigma_{SC}^{V}\right).L_{SC}.C_{SC}^{V}-S_{LU}.\left(1-\sigma_{SC}^{I}\right).L_{SC}.C_{SC}^{I}+{CL}_{up}^{SC}.\left(1-FR\right).C_{SC}^{E\_Bound}-{CL}_{up}^{SC}.C_{I}^{SC}-K_{SC}.C_{I}^{SC})/V_{SC}^{I}$$

Please refer to Supplementary Tables S1 and S2 for description and units of the symbols used in the above equations.

### 2.5. Model Parameterization and Estimation

All model parameters apart from pinocytosis rate (CL_up_) and non-specific degradation rate of unbound antibody (k_deg_) are the same as those used for humans by Shah and Betts [9]. The central plasma volume and blood cell volume used in the current model are equal to the total blood volume subtracted from the vascular volume present in the tissues. Thus, the central plasma volume and blood cell volume used in the current model are 1412 and 1155 mL. To estimate inter-antibody variability similar to inter-individual modeling, each antibody was treated as an individual. Population as well as antibody-specific ${Cl}_{up}$ and $K_{deg}$ estimates and inter-antibody variability of these parameters were obtained by fitting the model to the multiple-dose-level IV pharmacokinetic data of the 46 mAbs in Monolix 2021R1.

For the SC model, the skin was divided into a subcutaneous compartment and the rest of the skin compartment in order to account for the region of skin where the subcutaneous dose is administered. Since subcutaneous doses are usually given as ~2 mL injections, we considered the interstitial sub-compartment of the SC compartment to be 2.25 mL as the SC dose is administered in the interstitial region of the skin. The other sub-compartment volumes of the SC compartment were then calculated by maintaining the ratio between the sub-compartments to be the same as that in the skin. The plasma, blood cell flow, and lymph flow rates for the SC compartment were also scaled down and calculated based on the new SC tissue volume from the original skin compartment. The parameters related to the Skin and SC compartments in both IV as well as SC models have been provided in Table 1. Antibody-specific CL_up_ and k_deg_ estimates obtained previously by fitting IV data were fixed (used as regressors in Monolix) for the sixteen mAbs that had both IV and SC pharmacokinetic data available. The SC model was then fitted to their SC PK profiles, and population, as well as individual absorption-related parameters (k_SC_ and S_LU), were estimated along with their inter-antibody variability. The Stochastic Approximation Expectation-Maximization (SAEM) algorithm in Monolix 2021R1 was used for nonlinear mixed effects modeling, and standard errors were computed with the Fisher information matrix. The combined error model was specified, and it was assumed that CL_up_, k_deg_, k_sc,_ and S_LU parameters follow log-normal distributions.

To quantitatively compare observed and model-predicted PK profiles, the % prediction error for AUC_0-t_ was calculated using the equation below.

$$\%PE=1-\frac{\left|{AUC}_{pred}-\left.{AUC}_{obs}\right|\right.}{{AUC}_{obs}}\times 100$$

where ${AUC}_{pred}$ refers to ${AUC}_{0-t}$ for the model-predicted PK profile and ${AUC}_{obs}$ refers to ${AUC}_{0-t}$ for the observed PK profile. For antibodies with PK data at multiple dose levels, the median of %PE values for different dose levels was calculated.

### 2.6. Sensitivity Analysis

In order to better understand the effect of two novel parameters introduced to capture the PK of antibodies following SC administration, a local sensitivity analysis was performed, where the effect of changes in S_LU and ksc on the PK behavior of mAbs was assessed. The parameters were altered ±50%, and the percent change in the area under the antibody exposure curve (AUC) was calculated using the following equation:

$$\%\;Change\;in\;AUC=\frac{\left({AUC}_{\pm 50\%}-{AUC}_{orig}\right)}{{AUC}_{orig}}$$

where ${AUC}_{\pm 50\%}$ refers to the AUC obtained after changing the parameters by 50%, and ${AUC}_{orig}$ refers to the AUC with model-estimated population parameters.

### 2.7. Monte Carlo Simulations

The established PBPK models were used to simulate a range of possible PK profiles for mAbs that exhibit linear pharmacokinetics post intravenous and subcutaneous administration. The Monte Carlo simulations were conducted for 1000 patients using the Ubiquity package in R [14]. The profiles were simulated with estimated population parameter values of CL_up_, k_deg_, k_SC_, and S_LU; the inter-antibody variability that was estimated for these parameters was also incorporated. To validate the predicted window of SC PK profiles, we overlay SC PK data for nine mAbs on the predicted population simulation. These mAbs were not used for developing the model. Several of the mAbs used for validation had pharmacokinetic data at multiple dose levels. Thus, all PK data for each antibody were dose-normalized to their highest dose profile and overlaid on population simulations conducted for the highest dose to validate a priori model-predicted antibody PK following SC administration.

## 3. Results

### 3.1. mAb Clinical PK Data

PK data were collected for a total of 143 mAbs out of which 75 were FDA-approved and 68 others were clinically tested. Among the mAbs in our database, 55 exhibited linear PK and were used for further analysis. Panobacumab (IgM) and Suvratoxumab (Fc-modified) showed linear PK but were excluded from model development as they have substantially different binding affinities for FcRn compared to the rest of the IgG molecules. A total of 44 mAbs with IV data were used to establish the IV popPBPK model, and 16 mAbs with both IV and SC data were used to establish the SC popPBPK model. Nine mAbs that lacked IV data but had SC data were used to validate the established popPBPK model. A summary of all datasets used in the development of the popPBPK model is provided in Table 2. Additional information on the antibody datasets can be found in Supplementary Table S3.

**Figure 3:**
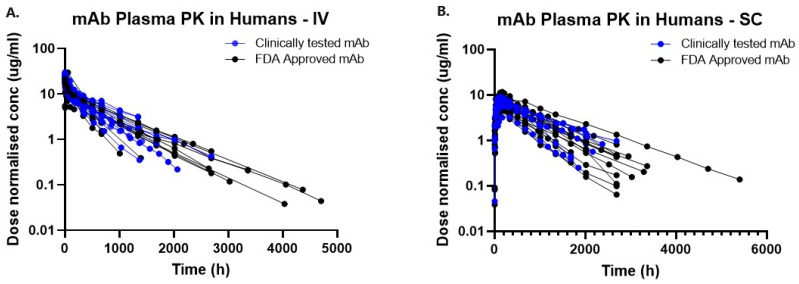
Highest dose PK profiles obtained for each antibody dose normalized to 1 mg/kg. Clinically tested and FDA-approved mAbs are plotted in blue and black. No clear distinctions in their pharmacokinetics were observed based on their ‘status of approval’. (**A**) mAb Plasma PK in Humans—IV. (**B**) mAb Plasma PK in Humans—SC.

IV and SC PK profiles of all mAbs were dose-normalized to 1 mg/kg and overlaid on each other to visually inspect inter-antibody variability and its influence on FDA approval. In the absence of TMDD and immunogenicity, moderately diverse PK profiles were observed post IV and SC administration. There was an approximately 6-fold difference in dose-normalized antibody exposures (AUCs), with AUC values falling in the range of 1970–12,900 μg/mL·h. No clear distinction in pharmacokinetics was observed between mAbs that were FDA-approved and those that were solely clinically tested (Figure 3).

### 3.2. popPBPK Model Fitting and Parameter Estimation

**Figure 4:**
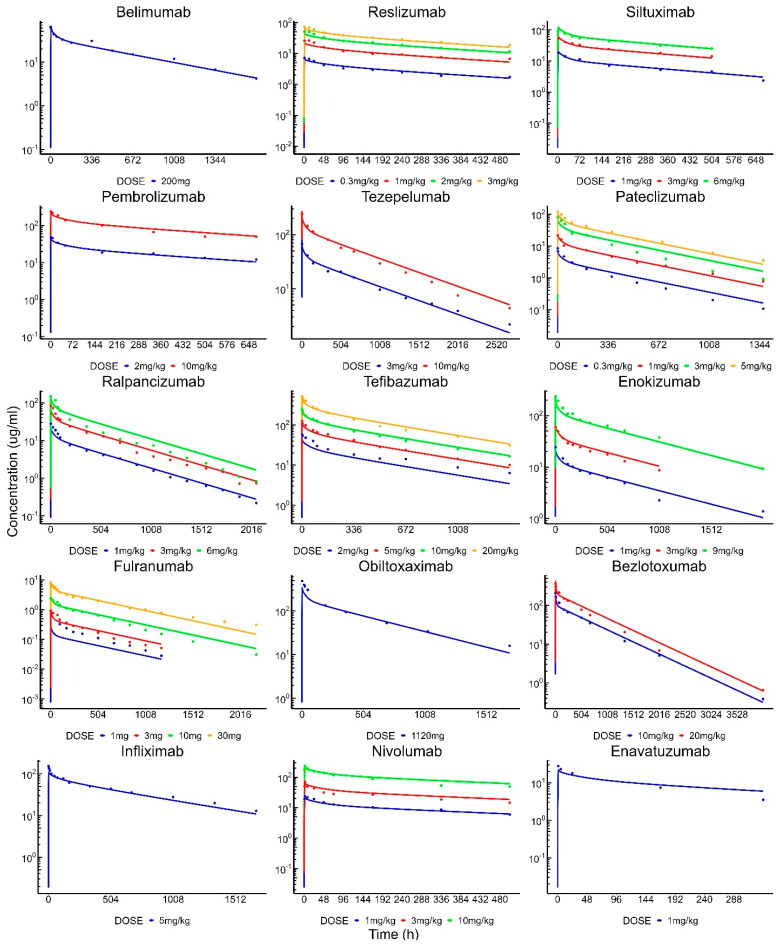

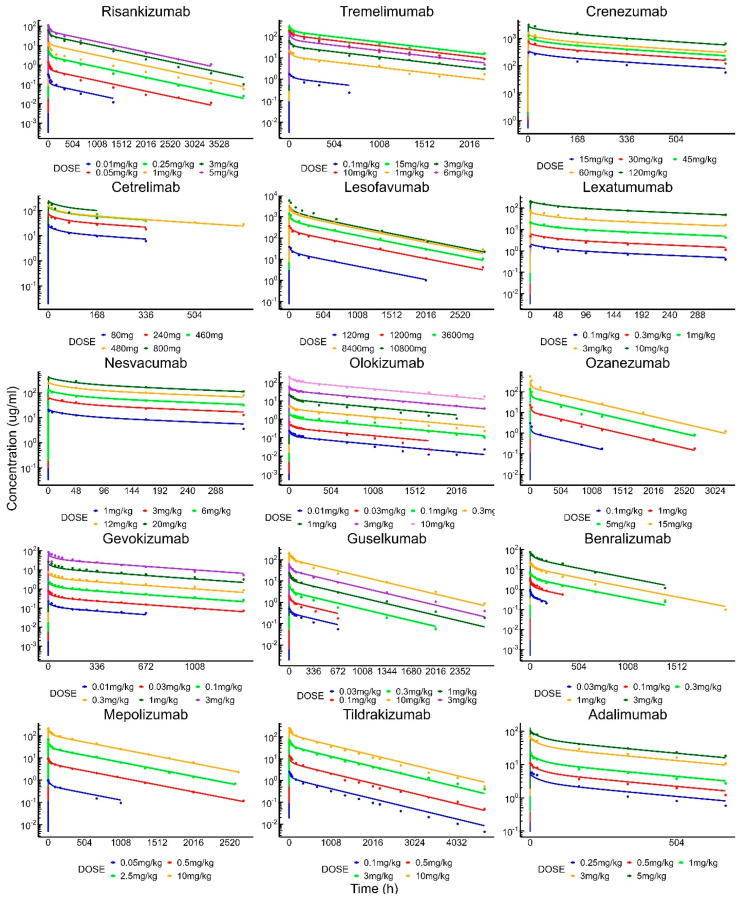

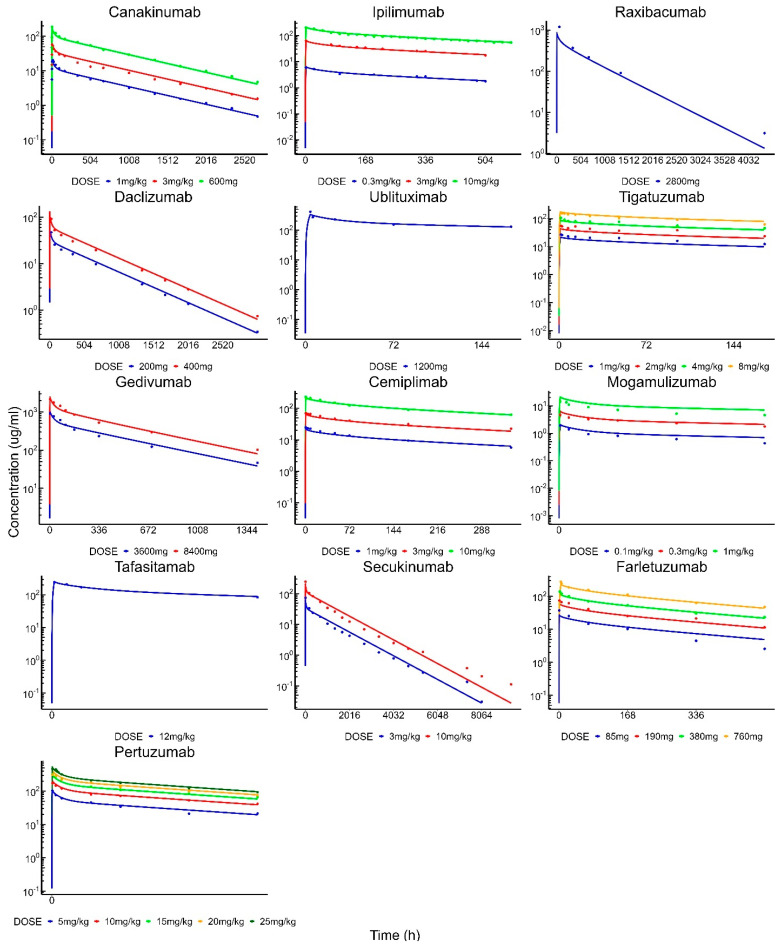
Clinical PK of IV-administered mAbs with individual model fittings. Solid dots represent observed data and solid lines represent model-fitted profiles. Each dose is plotted with a different color for mAbs with multiple dose-level data.

**Figure 5:**
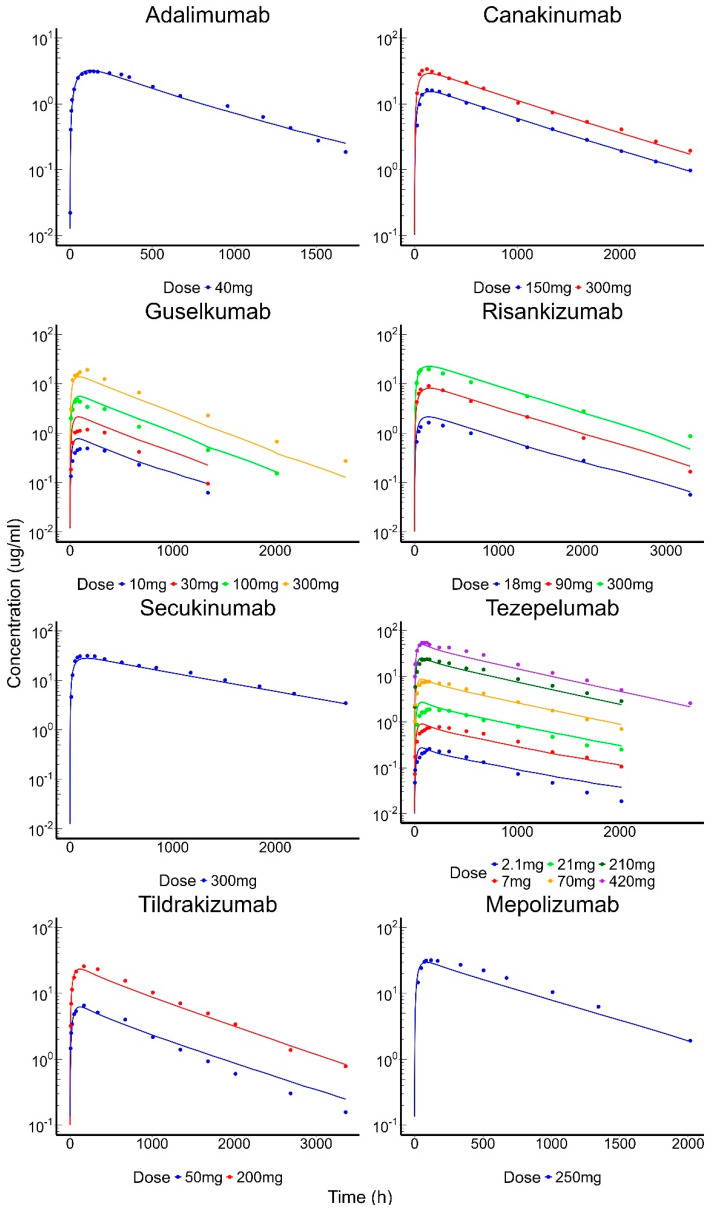

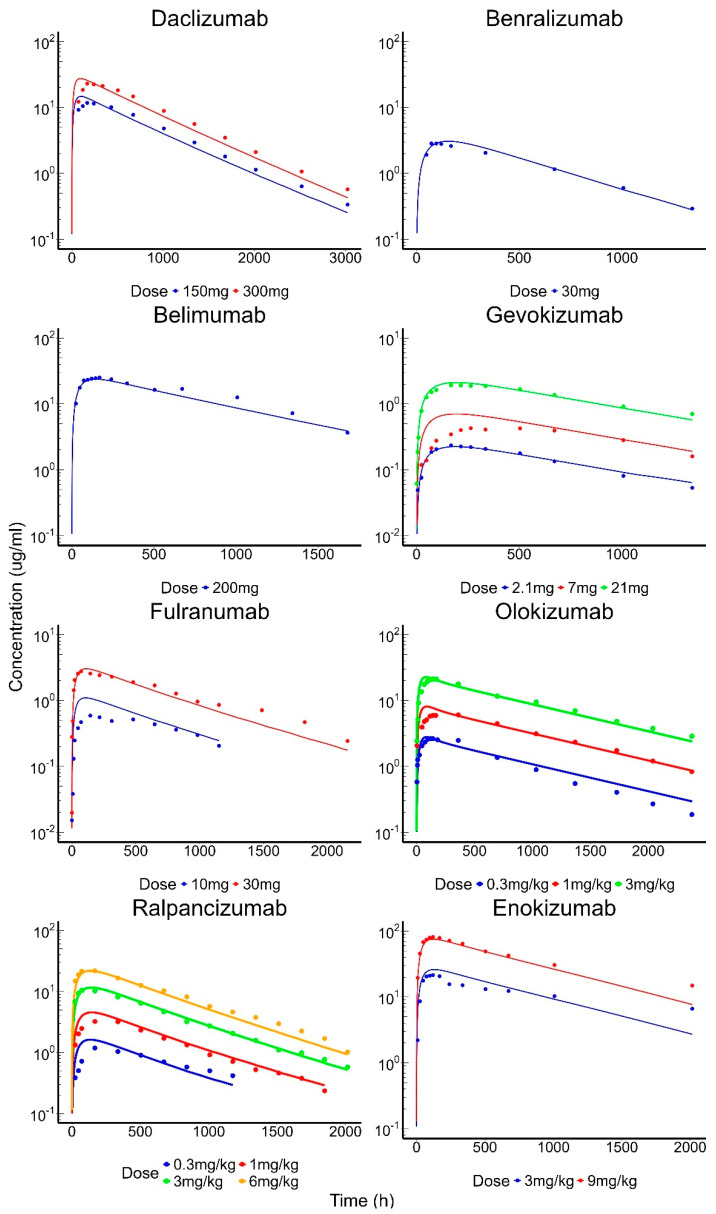
Clinical PK of SC-administered mAbs with individual model fittings. Solid dots represent observed data and solid lines represent model-fitted profiles. Each dose is plotted with a different color for mAbs with multiple dose-level data.

The IV PBPK model was adapted from Shah and Betts [9], and all original parameter values, apart from CL_up_ and k_deg_, were retained. In order to capture the inter-antibody variability in the pharmacokinetics of linear mAbs, variability was added to the pinocytic uptake (CL_up_) and non-specific degradation (k_deg_) parameters. In a prior local sensitivity analysis of the PBPK model [9], both k_deg_ and CL_up_ were found to be drug-specific sensitive parameters in the model. The popPBPK model was able to adequately capture the PK of all 43 mAbs, as shown in Figure 4. The %PE for all antibodies was below 30%, individual %PE are provided in Table 3. The population PK parameters and their inter-antibody variability were estimated with reasonable confidence (<20% CV). The estimated CL_up_ (pop), $\omega_{Clup}$, k_deg_ (pop), and $\omega_{Kdeg}$ were 0.32 L/h/L, 73%, 26.1 $h^{-1}$, and 46%. In order to capture the SC data, the skin compartment was bifurcated into an ‘SC compartment’, which represents the area of skin where the dose is administered and the ‘rest of the skin compartment’. Further variability was assigned to the lymphatic uptake process (S_LU) and local degradation (k_sc_) to account for reported heterogeneity in the antibody absorption process. The SC popPBPK model was able to fit the model to the data well (Figure 5), with %PE values < 30% (Table 4), and estimated the population parameters k_sc_ and S_LU with reasonable confidence (66% and 14% RSE). The model estimates for k_sc_ (pop), $\omega_{Ksc}$, S_LU (pop), and $\omega_{S\_LU}$ were 0.0015 $h^{-1}$, 193%, 0.54, and 49%.

**Figure 6:**
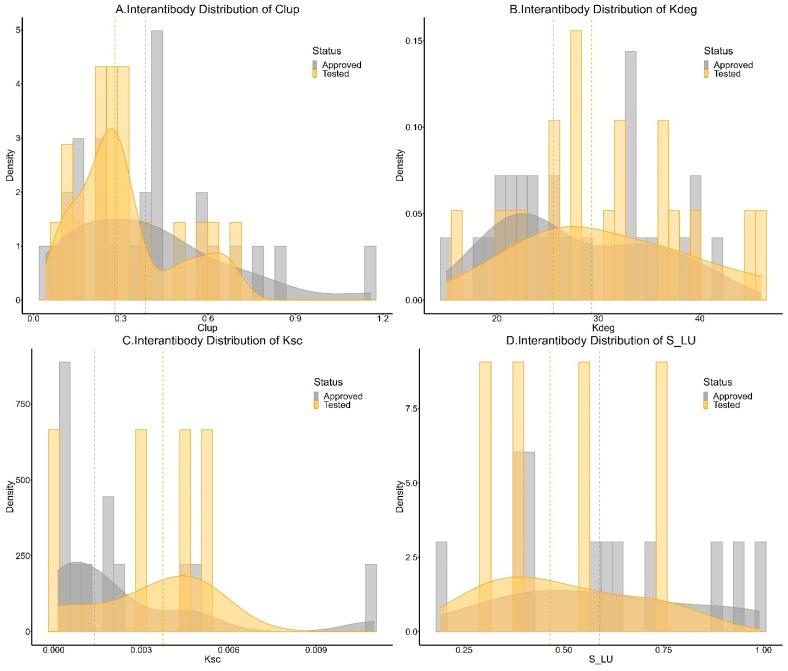
Distribution of parameters (**A**) CL_up_, (**B**) k_deg_, (**C**) k_SC_, and (**D**) S_LU for FDA-approved and clinically tested mAbs. Gray and yellow dotted lines indicate the distribution medians for FDA-approved and clinically tested mAbs.

Based on individual parameter estimates obtained in the model development process, distributions of possible parameter values in the clinic were obtained; these are shown in Figure 6. The distributions of parameters were categorized based on their status of approval to identify any potential differences. FDA-approved mAbs had a median CL_up_ value of 0.39 L/h/L whereas that of the clinically tested mAbs was 0.28 L/h/L. The approved and clinically tested mAbs had similar median k_deg_ estimates of 25.5 and 29.3 $h^{-1}$. Thus, no clear distinction was inferred in the disposition of FDA-approved and clinically tested mAbs. FDA-approved mAbs showed 2.7 times lower median local degradation (0.0014 vs. 0.0038 $h^{-1}$) and marginally greater lymphatic uptake (0.59 vs. 0.47), suggesting a better absorption profile of FDA-approved mAbs.

### 3.3. Sensitivity Analysis

**Figure 7:**
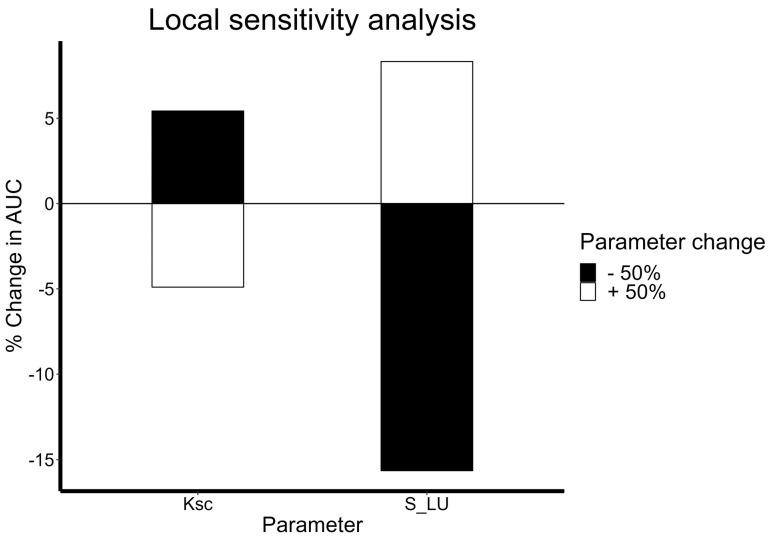
Local sensitivity analysis of absorption-related parameters k_sc_ and S_LU. Percent change and the direction of change in AUC with an increase or decrease of 50% in parameter values are shown. A greater absolute value indicates a more sensitive parameter, and S_LU is a more sensitive term than k_sc_.

A local sensitivity analysis helps identify how sensitive model predictions are to change with certain parameter values. It also helps explore how much individual processes influence mAb pharmacokinetics and the direction of their effects. Here, a sensitivity analysis was performed on S_LU and k_SC_, which refer to the lymphatic uptake scaling factor and rate of local degradation at the drug administration site. Greater sensitivity to a certain parameter would lead to a greater absolute value of percent change in AUC. The model was found to be significantly sensitive to both absorption-related parameters, with S_LU being the more sensitive parameter (Figure 7). The T_max_ of the PK profiles was directly proportional to lymphatic uptake, thus highlighting the importance of this process in the rate and extent of absorption. Additionally, S_LU was directly proportional to drug exposure, with greater lymphatic uptake associated with more drugs in the system and greater net exposures. On the contrary, k_SC_ was inversely proportional to drug exposure as expected. Greater rates of local degradation would lead to decreased bioavailability of drugs and thus lower net exposures.

### 3.4. Monte Carlo Simulations and Model Validation

**Figure 8:**
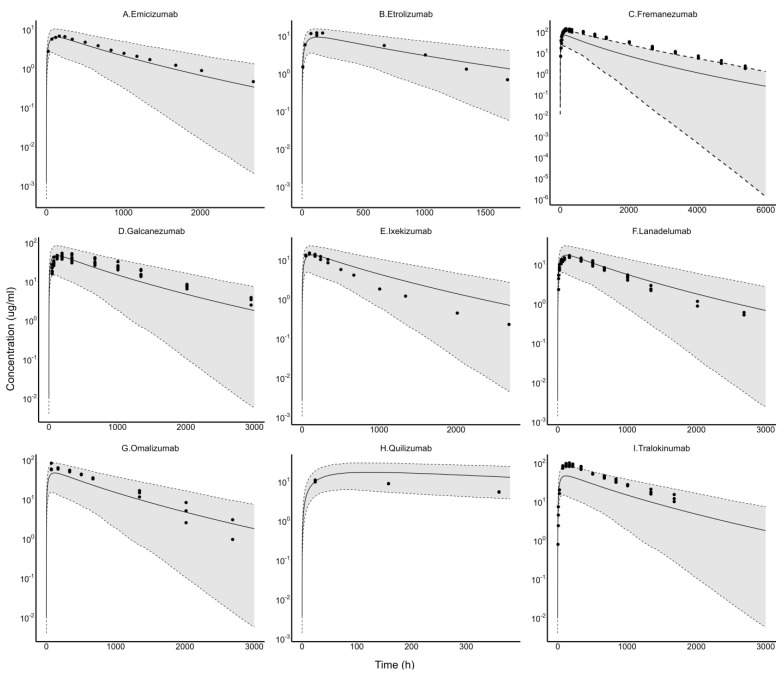
popPBPK model-predicted window and observed concentration–time profiles for validation mAbs following SC injection. Data were dose-normalized to the highest available dose, and simulations were performed for the same dose. The mAbs shown are: (**A**) Emicizumab (70 mg), (**B**) Etrolizumab (105 mg), (**C**) Fremanezumab (900 mg), (**D**) Galcanezumab (600 mg), (**E**) Ixekizumab (160 mg), (**F**) Lanadelumab (210 mg), (**G**) Omalizumab (600 mg), (**H**) Quilizumab (210 mg), and (**I**) Tralokinumab (600 mg).

Monte Carlo simulations were conducted to simulate a prediction window of PK profiles for linear mAbs post-SC dosing. SC PK profiles of emicizumab, etrolizumab, fremanezumab, galcanezumab, ixekizumab, lanadelumab, omalizumab, quilizumab, and tralokinumab were used to validate the simulated prediction window. Data for multiple dose levels was available for fremanezumab, galcanezumab, omalizumab, tralokinumab, and lanadelumab, and Monte Carlo simulations were carried out for the highest dose available for each of these mAbs. All observed PK data were normalized to the highest dose and overlaid onto the simulations (Figure 8). In general, all SC PK profiles fall within the prediction window simulated from the popPBPK model, thus providing confidence in the use of the model to *a priori* predict the range of clinical PK for mAbs exhibiting linear clearance.

## 4. Discussion

In the past twenty years, the mAb drug class has grown exponentially, with more than 1000 mAbs currently in the clinical pipelines of pharmaceutical companies, and redefined targeted therapy. The field has expanded beyond cancer, infections, and immunological disorders to neurological diseases [68], rare genetic disorders [69], migraines [70], osteoporosis [71], eye disorders [72], and many others. Studies have reported relatively similar pharmacokinetics of mAbs with linear clearance regardless of their targets owing to their similar size and structure [7]. This is highly advantageous as it can help predict the clinical PK behavior of certain mAbs. In order to make informed predictions, it is necessary to not only understand general PK behavior but also the source and extent of clinical PK variability. Whereas several studies have attempted to identify the variability source and build mathematical relations to predict PK [73,74,75], there have been few efforts to characterize the extent of this variability [7,8]. Additionally, previous analyses have been limited to general NCA or empirical compartmental modeling parameters, like central volume, peripheral volume, and clearance, thus providing limited mechanistic insight into variability in individual processes involved in antibody absorption, distribution, and elimination.

In this study, available clinical PK data of all approved and clinically tested mAbs were collected and used to build a popPBPK model to characterize the general behavior as well as inter-antibody pharmacokinetic variability of mAbs with linear PK. The use of a popPBPK model to characterize this variability accommodated the mathematical representation of the human body and various physiological processes involved in antibody PK and assigned variability terms to discrete processes. This enabled the exploration of the distribution of drug-specific parameters like CL_up_, k_deg_, S_LU, and k_SC_ and the prediction of the clinical PK of mAbs with linear disposition.

Subcutaneous administration of mAbs is becoming increasingly popular owing to its ease of administration, increased patient adherence, and reduced burden on the healthcare system. Despite extensive interest in the field, the development of SC mAbs is relatively slow as the absorption is highly variable and there are several gaps in understanding of factors regulating the absorption process [76]. Furthermore, substantial differences in SC mAb absorption in preclinical species and humans have been reported [77]. In such scenarios, in silico tools like PBPK models are very useful and have been used to explore determinants of antibody SC absorption [78]. In order to characterize the SC absorption of mAbs, an SC compartment was separated from the skin compartment in the popPBPK model. SC-administered mAbs undergo local degradation at the site of injection [79], and to account for this, a first-order rate constant of degradation was added at the site of injection (k_SC_). A scaling factor (S_LU) was also multiplied by the lymphatic uptake process of mAbs from the site of injection to represent the differences in the local distribution of mAbs at the injection site stemming from different physiochemical properties and the variability in antibody escape from the extracellular matrix [80].

The IV and SC PBPK models were able to capture most of the PK data. The model was unable to adequately capture the PK profiles of a few mAbs at select dose levels. For example, the model was unable to characterize the initial phase of fulranumab PK following a 1 mg dose. Since the model was able to capture fulranumab PK at other dose levels successfully, we hypothesize that this issue may have been caused by some error in either reporting the PK data or error in data collection after the 1 mg dose. Similarly, the model encountered difficulty fitting the PK profiles of Olokizumab at 0.01 mg/kg and Tefibazumab at 2 mg/kg, where PK concentrations increase at later time points post a single intravenous dose. The increase in concentrations at later time points could be attributed to mAb lymphatic recirculation [81], measurement error, or errors in digitizing mean data. Since increased concentrations were only observed at the lowest antibody dose levels in both cases, where concentrations at later time points might be close to the limit of quantification of the analytical method, it is possible that the error can be associated with data collection or reporting issues. The population parameters for CL_up_, k_deg_, and S_LU and their BDV (between drug variability) were estimated with reasonable confidence (<20 CV%). The k_SC_ parameter was estimated with a greater RSE (66%). This could be because the parameter is relatively less sensitive. A local sensitivity analysis was performed to assess the dynamics of SC absorption and the impact of variability in local degradation and lymphatic uptake. The process of lymphatic uptake was found to be more sensitive than local degradation. These results align with other studies that have reported lymphatic uptake to be the most important route for absorption of subcutaneously administered mAbs [78,80].

Currently, only around 20% of the mAbs entering clinical trials get approved [82]. The identification of a distinct favorable property profile of approved mAbs would represent a strategic advantage and help to establish ‘benchmarks’ of clinical success. However, there were no differences in the IV and SC PK profiles for approved vs. not yet approved mAbs, suggesting that poor pharmacokinetics is an unlikely source of antibody–drug attrition. These results must be interpreted cautiously as the database might be biased with PK data from mAbs with desirable PK properties, which are more likely to be published. The parameter distributions of the model-estimated parameters CL_up_, k_deg_, k_SC,_ and S_LU provide an additional opportunity to assess the properties of approved drugs and compounds under development. Estimated CL_up_ values ranged from lower values like 0.04 for infliximab to higher values like 1.16 for bezlotoxumab. Whereas most mAbs fell within the 0.04–0.83 range, bezlotoxumab showed a greater rate constant of pinocytosis. The median CL_up_ for approved mAbs (0.39) was marginally greater than that of clinically tested mAbs (0.28). Estimated K_deg_ parameters fell in the range of 15–46 $h^{-1}$ with similar median k_deg_ values of 25.5 and 29 $h^{-1}$ for approved and clinically tested mAbs. No clear distinctions in CL_up_ and k_deg_ distribution between FDA-approved and clinically tested mAbs were observed. The estimated k_SC_ and S_LU parameters ranged from 0.00012 to 0.0051 $h^{-1}$ and 0.19–0.99. Guselkumab had a significantly greater local degradation rate constant (0.011) than the rest of the mAbs. The bioavailability of guselkumab is also reported to be 49% [83], which is less than the normal range of 60–80% reported for other marketed mAbs [84]. One hypothesis is that faster local degradation at the site of injection could be responsible for the lower SC bioavailability of guselkumab. The FDA-approved mAbs had smaller rate constants of local degradation and a marginally greater rate constant for lymphatic uptake, suggesting that approved mAbs might have better absorption profiles than the other tested mAbs. Since only four clinically tested mAbs and twelve approved mAbs were examined, data from more mAbs are needed to confirm this conclusion. Nonetheless, the popPBPK model developed here not only provides simulations for the mean PK profiles but also incorporates inter-antibody variability seen in the clinic to better support antibody development. While several parameters like ${Cl}_{up}$ and plasma volume have been updated in the current model, the predicted PK profile matches well with a previously published PBPK model for antibodies and provides better characterization of the alpha phase of the PK profile (Figure S1).

The primary value of the established popPBPK model lies in its ability to simulate a prediction window at different dose levels for mAbs with linear clearance. We validated the model-based prediction window by overlaying digitized SC concentration–time data from nine different mAbs, which could not be used for model development owing to a lack of IV data. The model was able to *a priori* predict the range within which the PK profiles of all validation mAbs fell. This prediction window provides a general idea of what to expect in the clinic post IV or SC administration for mAbs with linear PK. This prior knowledge could help identify optimal doses for clinical trials, consolidate trial sample size, and expedite the time to drug approval. Additionally, molecules with substantial deviations from this general mAb PK profile during the drug discovery and development process could be flagged for further analysis. The parameter distributions also provide information about the variability in individual processes of antibody pharmacokinetics.

In this study, we observed considerable variability in the clinical PK of mAbs with linear PK, leading to a relatively broad prediction window for antibody PK. While previous research has established that the size of protein therapeutics significantly influences their PK properties [85], our findings suggest that antibodies of similar size can exhibit substantially different PK profiles. This variability may be attributed to other physicochemical properties, such as charge, glycosylation pattern, and hydrophobicity [86]. Multiple studies have been conducted to identify relationships between the physicochemical properties of antibodies and their PK, yielding promising results [73,74]. However, further quantitative structure–pharmacokinetic relationship (QSPKR) studies are required to elucidate some of this variability and help narrow the prediction window. Future research in this area will be essential for a more comprehensive understanding of the factors influencing variability in antibody PK.

The final popPBPK model does not account for diverse study populations and analysis methods. Since mean concentration–time profiles from different clinical studies conducted in diverse populations were utilized, the variability observed in digitized PK profiles might be inflated possibly owing to distinct study populations, analysis methods, sample collection techniques, and patient co-medications as well. While the current study facilitates prediction of the pharmacokinetics for mAbs that exhibit linear PK, there is a need to incorporate target-mediated drug disposition and its associated variability in the model to be able to successfully predict the clinical PK for all mAbs. Further analysis is required to associate sources of variability like different physicochemical properties to explain some of the inter-antibody variability and make more accurate predictions.

In summary, a popPBPK model for IV- and SC-administered mAbs using a comprehensive clinical PK dataset, which characterizes the extent of inter-antibody variability in pharmacokinetics seen in the clinic, has been developed. The established model enables predictions of IV and SC PK for mAbs with linear PK before conducting clinical trials and could possibly assist with preclinical-to-clinical translation and ‘first-in-human’ dose determination.

## Figures and Tables

**Table 1:** SC tissue compartment volumes and flows in the popPBPK model.

Tissue Volumes (mL)	Skin (SC)	SC (SC)
Total volume	3401	6.82
Plasma volume	127.2	0.25
Blood cell volume	104.1	0.21
Interstitial volume	1123	2.25
Endosomal volume	17.01	0.03
Cellular volume	2031	4.07
**Tissue flow (mL/h)**		
Plasma flow	11,600	23.25
Blood cell flow	9493	19.02

**Table 2:** List of all the antibodies used for the development of the PopPBPK model.

ID	Name	IV Doses	SC Doses	Status	Reference
Datasets used for IV and SC model development					
1	Adalimumab	(0.25, 0.5, 1, 3, and 5) mg/kg	40 mg	Approved	[15]
2	Belimumab	200 mg	200 mg	Approved	[16]
3	Benralizumab	(0.03, 0.1, 0.3, 1 and 3) mg/kg	30 mg	Approved	[17,18]
4	Canakinumab	(1 and 3) mg/kg and 600 mg	(150, 300) mg	Approved	[19]
5	Daclizumab	200 and 400 mg	(150, 300) mg	Approved	[20]
6	Enokizumab	(0.3, 1, 3 and 9) mg/kg	(3, 9) mg/kg	Tested	[21]
7	Fulranumab	(1, 3, 10 and 30) mg	(10, 30) mg	Tested	[22]
8	Gevokizumab	(0.01, 0.03, 0.1, 0.3, 1 and 3) mg/kg	(2.1, 7, 21)mg	Approved	[23]
9	Guselkumab	(0.03, 0.1, 0.3, 1, 3, and 10) mg/kg	(10, 30, 100, 300) mg	Approved	[24]
10	Mepolizumab	(0.05, 0.5, 2.5 and 10) mg/kg	250 mg	Approved	[25]
11	Olokizumab	(0.01, 0.03, 0.1, 0.3, 1, 3 and 10) mg/kg	(0.3, 1, 3) mg/kg	Tested	[26]
12	Ralpancizumab	(1, 3 and 6) mg/kg	(0.3, 1, 3, 6) mg/kg	Tested	[27]
13	Risankizumab	(0.01, 0.05, 0.25, 1,3 and 5) mg/kg	(18, 90, 300) mg	Approved	[28,29]
14	Secukinumab	(3 and 10) mg/kg	300 mg	Approved	[30,31]
15	Tezepelumab	(3 and 10) mg/kg	(2.1, 7, 21, 70, 210, 420) mg	Approved	[32]
16	Tildrakizumab	(0.1, 0.5, 3 and 10) mg/kg	(50, 200) mg	Approved	[33]
Datasets used only for IV model development					
17	Bezlotoxumab	(10 and 20) mg/kg	-	Approved	[34]
18	Cemiplimab	(1, 3 and 10) mg/kg	-	Approved	[35]
19	Cetrelimab	(80, 240, 460, 480 and 800) mg	-	Tested	[36]
20	Crenezumab	(15, 30, 45, 60 and 120) mg/kg	-	Tested	[37]
21	Enavatuzumab	1 mg/kg	-	Tested	[38]
22	Farletuzumab	(85, 190, 380 and 760) mg	-	Tested	[39]
23	Gedivumab	(3600 and 8400) mg	-	Tested	[40]
24	Infliximab	5 mg/kg	-	Approved	[41]
25	Ipilimumab	(0.3, 3 and 10) mg/kg	-	Approved	[42]
26	Lesofavumab	(120, 1200, 3600, 8400 and 10,800) mg	-	Tested	[43]
27	Lexatumumab	(0.1, 0.3, 1, 3 and 10) mg/kg	-	Tested	[44]
28	Mogamulizumab	(0.1, 0.3 and 1) mg/kg	-	Approved	[45]
29	Nesvacumab	(1, 3, 6, 12 and 20) mg/kg	-	Tested	[46]
30	Nivolumab	(1, 3 and 10) mg/kg	-	Approved	[47]
31	Obiltoxaximab	1120 mg	-	Approved	[48]
32	Ozanezumab	(0.1, 1, 5 and 15) mg/kg	-	Tested	[49]
33	Pateclizumab	(0.3, 1, 3 and 5) mg/kg	-	Tested	[50]
34	Pembrolizumab	(2 and 10) mg/kg	-	Approved	[51]
35	Pertuzumab	(5, 10, 15, 20 and 25) mg/kg	-	Approved	[52]
36	Raxibacumab	2800 mg	-	Approved	[53]
37	Reslizumab	(0.3, 1, 2 and 3) mg/kg	-	Approved	[54]
38	Siltuximab	(1,3 and 6) mg/kg	-	Approved	[55]
39	Tafasitamab	12 mg/kg	-	Approved	[56]
40	Tefibazumab	(2,5,10 and 20) mg/kg	-	Tested	[57]
41	Tigatuzumab	(1,2,4 and 8) mg/kg	-	Tested	[58]
42	Tildrakizumab	(0.1, 0.5, 3 and 10) mg/kg	-	Approved	[33]
43	Ublituximab	1200 mg	-	Approved	[59]
Datasets used for validation					
44	Emicizumab	-	1 mg/kg	Approved	[60]
45	Etrolizumab	-	105 mg	Tested	[61]
46	Fremanezumab	-	(225, 675, 900) mg	Approved	[62]
47	Galcanezumab	-	(1, 5, 25, 75, 200, 600) mg	Approved	[63]
48	Ixekizumab	-	(80, 160) mg	Approved	[64]
49	Lanadelumab	-	(0.1, 0.3, 1, 3) mg/kg	Approved	[65]
50	Omalizumab	-	(75, 300, 600) mg	Approved	FDA
51	Quilizumab	-	(70,210) mg	Tested	[66]
52	Tralokinumab	-	(150, 300, 600) mg	Approved	[67]

**Table 3:** Median percent prediction error (%PE) for quantitative comparison of observed and model-generated data for intravenously administered antibodies.

ID	Antibody	Median %PE	ID	Antibody	Median %PE
1	Belimumab	7.5	23	Olokizumab	12.8
2	Reslizumab	5.9	24	Ozanezumab	7.9
3	Siltuximab	6.0	25	Gevokizumab	8.3
4	Pembrolizumab	5.0	26	Guselkumab	16.1
5	Tezepelumab	3.3	27	Benralizumab	4.8
6	Pateclizumab	11.8	28	Mepolizumab	4.2
7	Ralpancizumab	2.8	29	Tildrakizumab	4.9
8	Tefibazumab	6.3	30	Adalimumab	7.3
9	Enokizumab	9.3	31	Canakinumab	3.9
10	Fulranumab	6.8	32	Ipilimumab	3.0
11	Obiltoxaximab	12.9	33	Raxibacumab	28.4
12	Bezlotoxumab	6.7	34	Daclizumab	8.7
13	Infliximab	7.2	35	Ublituximab	2.6
14	Nivolumab	14.2	36	Tigatuzumab	19.3
15	Enavatuzumab	5.0	37	Gedivumab	3.3
16	Risankizumab	12.4	38	Cemiplimab	3.4
17	Tremelimumab	13.8	39	Mogamulizumab	22.7
18	Crenezumab	6.5	40	Tafasitamab	4.9
19	Cetrelimab	11.2	41	Secukinumab	5.6
20	Lesofavumab	8.2	42	Farletuzumab	9.6
21	Lexatumumab	13.1	43	Pertuzumab	5.6
22	Nesvacumab	5.1			

**Table 4:** Median percent prediction error (%PE) for quantitative comparison of observed and model-generated data for subcutaneously administered antibodies.

ID	Antibody	Median % PE	ID	Antibody	Median % PE
1	Adalimumab	3.6	9	Daclizumab	5.0
2	Canakinumab	2.3	10	Benralizumab	6.1
3	Guselkumab	28.9	11	Belimumab	13.7
4	Risankizumab	10.7	12	Enokizumab	7.2
5	Secukinumab	7.6	13	Gevokizumab	2.7
6	Tezepelumab	11.5	14	Fulranumab	28.1
7	Tildrakizumab	8.0	15	Olokizumab	4.12
8	Mepolizumab	19.8	16	Ralpancizumab	10.69

## Data Availability

All the data used for this publication come from the public domain, and the source of each dataset is cited in the publication.

## Supplementary Materials

The following supporting information can be downloaded at: https://www.mdpi.com/article/10.3390/antib13030054/s1, Table S1: A glossary of parameters utilized in the antibody popPBPK model. ‘Sc’ refers to subcutaneous tissue. All other parameters are same as [9]; Table S2: Population parameters estimated from the antibody popPBPK model; Table S3: Information on Clinical studies; Figure S1: Comparison of current PBPK model with previously published PBPK model.
