# Supplementary material for: Inter-Antibody Variability in the Clinical Pharmacokinetics of Monoclonal Antibodies Characterized Using Population Physiologically Based Pharmacokinetic Modeling
Source: Antibodies (Basel). 2024 Jul 9;13(3):54. doi: 10.3390/antib13030054 (PMC11270311; doi:10.3390/antib13030054)

<u>Supplementary material</u>

Supplementary Table S1. A glossary of parameters utilized in the antibody popPBPK model. 'Sc' refers to subcutaneous tissue. All other parameters are same as [9].

Parameter	Definition	Unit
$V_{pl,} V_{BC,} V_{LN}$	Volume of central plasma, blood cell and lymph node compartment	L
$V_i^v, V_i^{BC}, V_i^E, V_i^I$	Volume of vascular, blood cell, endosomal and interstitial sub-compartment of tissue "i"	L
C_{pl}, C_{LN}, C_{BC}	mAb concentration in central plasma, lymph node and central blood cell compartment	M
$C_i^V, C_i^{BC},, C_i^I$	mAb concentration in plasma, blood cell and interstitial sub-compartment of tissue "i"	M
Q_i	Plasma flow to tissue "i"	L/h
L_i	Lymph flow to tissue "i"	
Q_i^{BC}	Blood cell flow to tissue "i"	L/h
σ_i^V, σ_i^I	Vascular and lymphatic reflection coefficient	-
FR	FcRn bound mAb fraction that is recycled to vascular space	-

Supplementary Table S2. Population parameters estimated from the antibody popPBPK model.

**Parameter**	**Definition**	**Model estimates (RSE%)**	**Units**
$Cl_{up}(pop)$	Population estimates for pinocytic uptake rate of mAbs per unit endosomal space.	0.32 (5.6)	L/h/L
ω_{Clup}	Inter-antibody variability in mAb pinocytic uptake rate per unit endosomal space.	73% (3.2)	-
$k_{deg}(pop)$	Population estimates for first order degradation rate constant of FcRN unbound mAb in endosomal space.	26.1 (11)	1/h
ω_{kdeg}	Inter-antibody variability in degradation rate constant of FcRN unbound mAb in endosomal space.	46% (17.6)	-
$S\_LU(pop)$	Scaling factor for lymphatic uptake of mAbs from interstitial space of subcutaneous tissue.	0.54 (14)	-
ω_{S_LU}	Inter-antibody variability in lymphatic uptake rate of mAbs from interstitial space of subcutaneous tissue.	49% (21.1)	-
k_{SC}	First order rate constant for local degradation of mAbs in subcutaneous tissue.	0.0015 (66)	1/h
ω_{ksc}	Interantibody variability in local degradation rate in subcutaneous tissue.	193% (24.7)	-

Supplementary Table S3. Information on Clinical studies.

ID	Name	Status	Phase of study	Data collected from Phase	Organising country	Year of study	Reference
1	Adalimumab	Approved		I	USA		[34]
2	Belimumab	Approved		I	USA and Canada		[35]
3	Benralizumab	Approved		I	USA<br>Germany	2008<br>2017	[36, 37]
4	Canakinumab	Approved		III	NCT00421226-Japan<br>NCT00619905-Germany,Netherlands,Switzerland<br>NCT00487708-USA,Belgium,France,Germany,India,Italy,Spain,Turkey,UK<br>NCT00465985-USA,France,Germany,India,Spain,UK	2012<br>2008<br>2010<br>2017	[38]
5	Daclizumab	Approved		I-III	NCT00390221-PhaseII,-Czech Republic	2011	[39]
6	Enokizumab	Tested	Phase II	I	NCT00192296-USA<br>NCT00116168-USA	2005<br>2013	[40]
7	Fulranumab	Tested	III	I	NCT02348879-2015-no info<br>NCT02318407-no info	2015<br>2014	[41]
8	Gevokizumab	Approved		I	NCT00541983-Switzerland	2010	[42]
9	Guselkumab	Approved		I	NCT00925574-USA	2017	[43]
10	Mepolizumab	Approved		III	UK	2011	[44]
11	Olokizumab	Tested	III	I	NCT01276119-Germany	2012	[45]
12	Ralpancizumab	Tested		I	NCT01720537- USA	2018	[46]
13	Risankizumab	Approved		I<br>III	NCT02596217-Japan,Korea<br>NCT03022045-Japan	2017<br>2021	[47, 48]
14	Secukinumab	Approved		I	USA	2011	[49, 50]
15	Tezepelumab	Approved		I	USA	2018	[51]
16	Tildrakizumab	Approved		I	USA	2017	[52]
17	Bezlotoxumab	Approved		I	Data taken from FDA review		[53]
18	Cemiplimab	Approved		I	NCT02383212-USA,Spain,Australia	2020	[54]
19	Cetrelimab	Tested	III	I	NCT02908906-US,Moldova,Poland,Russian Federation,Spain,Sweden, UK	2016	[55]
20	Crenezumab	Tested	III	Ib	NCT02353598-USA	2019	[56]
21	Enavatuzumab	Tested	I	I	USA		[57]
22	Farletuzumab	Tested	II	I	NCT01049061-Japan	2020	[58]
23	Gedivumab	Tested	II	II	NCT02293863-Belgium, Brazil,Bulgaria,Canada,Chile,France,Germany,HongKong,Hungary,Israel,Italy, Korea,Mexico,netherlands,NewZealanad,Peru,Poland,SA,Spain,Swedem,Taiwan,Ukraine,UK	2018	[59]
24	Infliximab	Approved		I			[60]
25	Ipilimumab	Approved		I			[61]
26	Lesofavumab	Tested		I	NCT02528903-Canada	2016	[62]
27	Lexatumumab	Tested	II	I			[63]
28	Mogamulizumab	Approved		I	NCT00888927-USA	2012	[64]
29	Nesvacumab	Tested	II	I	NCT01271972-USA,Canada	2015	[65]

30	Nivolumab	Approved		I	NCT02261285-Korea	2014	[66]
31	Obiltoxaximab	Approved		I<br>I<br>I<br>I<br>I	NCT00829582-USA<br>NCT01453907-USA<br>NCT01929226-USA<br>NCT01952444-USA<br>NCT01932242-USA	2017<br>2017<br>2013<br>2014<br>2014	[67]
32	Ozanezumab	Tested	II	I	NCT00875446-USA,ITALY,France,UK	2011	[68]
33	Pateclizumab	Tested	II	I	NCT00888745-USA,Hungary	2010	[69]
34	Pembrolizumab	Approved	III	I	NCT01840579.-NA	2020	[70]
35	Pertuzumab	Approved		I	Japan	2009	[71]
36	Raxibacumab	Approved		IV	NCT02339155-USA	2017	[72]
37	Reslizumab	Approved			Data taken from FDA review		[73]
38	Siltuximab	Approved		I			[74]
39	Tafasitamab	Approved		II	NCT02399085-USA,Czechia,Belgium,France,Germany,Hungary,Italy,Poland,Spain,UK	2016	[75]
40	Tefibazumab	Tested	II	I	USA		[76]
41	Tigatuzumab	Tested	II	I	USA		[77]
42	Tildrakizumab	Approved		I			[52]
43	Ublituximab	Approved		I	USA		[78]
44	Emicizumab	Approved		I	Chinese		[79]
45	Etrolizumab	Tested	III	I	NCT02996019-USA	2018	[80]
46	Fremanezumab	Approved		I	Japanese,Caucasians		[81]
47	Galcanezumab	Approved		I	Belgium	2012	[82]
48	Ixekizumab	Approved		I	NCT03073213-China	2019	[83]
49	Lanadelumab	Approved			Data taken from FDA review		[84]
50	Omalizumab	Approved			Data taken from FDA review		FDA
51	Quilizumab	Tested	II	II	NCT01582503-USA,Argentina,Belgium,Bulgaria,Canada,Germany,Hungary,Mexico,New Zealand,Peru,Russian Federation,Ukraine	2014	[85]
52	Tralokinumab	Approved		I	Japan		[86]

Supplementary Figure S1. Comparison of current PBPK model with previously published PBPK model. The plot displays PBPK model simulated human plasma concentrations post a 1mg/kg mAb dose. The black line represents median predicted concentrations while the shaded area corresponds to 90% CI. The red line represents simulations from previously published PBPK model from which the current model has been adapted. The black dots represent observed clinical data of antibodies dose normalized to 1mg/kg.

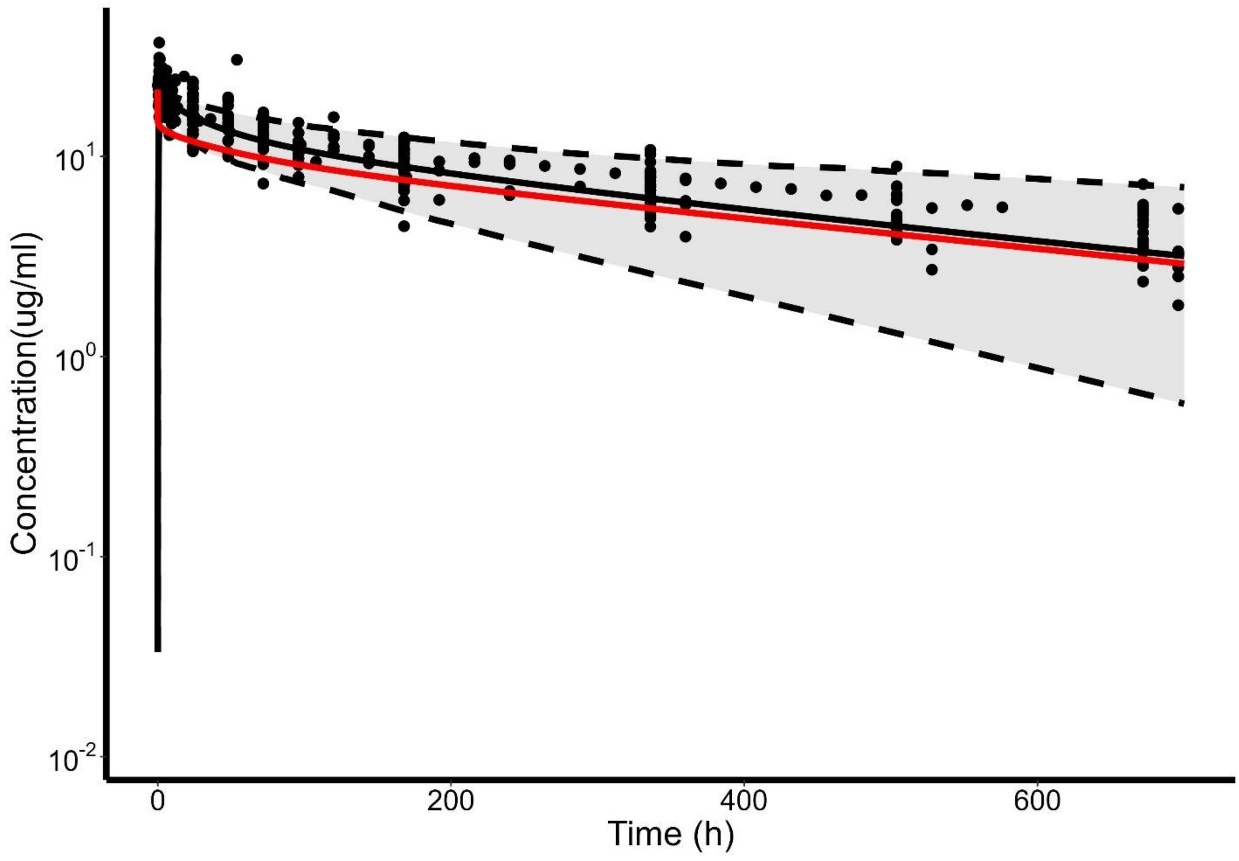

Supplement: Supplementary file 1 [file antibodies-13-00054-s001.zip › antibodies-3059681-supplementary.pdf]
